# Molecular Hydrogen Positively Affects Physical and Respiratory Function in Acute Post-COVID-19 Patients: A New Perspective in Rehabilitation

**DOI:** 10.3390/ijerph19041992

**Published:** 2022-02-10

**Authors:** Michal Botek, Jakub Krejčí, Michal Valenta, Andrew McKune, Barbora Sládečková, Petr Konečný, Iva Klimešová, Dalibor Pastucha

**Affiliations:** 1 Faculty of Physical Culture, Palacký University Olomouc, 771 11 Olomouc, Czech Republic; michal.botek@upol.cz (M.B.); michal.valenta@upol.cz (M.V.); barbora.sladeckova@upol.cz (B.S.); iva.klimesova@upol.cz (I.K.); 2 Research Institute for Sport and Exercise (UCRISE), University of Canberra, Bruce, ACT 2617, Australia; andrew.mckune@canberra.edu.au; 3 Discipline of Biokinetics, Exercise and Leisure Sciences, School of Health Sciences, University of KwaZulu-Natal, Durban 4041, South Africa; 4 Faculty of Health Sciences, Palacký University Olomouc, 775 15 Olomouc, Czech Republic; petr.konecny@upol.cz; 5 Clinic of Rehabilitation and Physical Medicine, University Hospital Ostrava, 708 52 Ostrava, Czech Republic; dalibor.pastucha@osu.cz

**Keywords:** hydrogen inhalation, COVID-19, health, fatigue, 6-min walking test, pulmonary function, oxygen saturation

## Abstract

Molecular hydrogen (H_2_) is potentially a novel therapeutic gas for acute post-coronavirus disease 2019 (COVID-19) patients because it has antioxidative, anti-inflammatory, anti-apoptosis, and antifatigue properties. The aim of this study was to determine the effect of 14 days of H_2_ inhalation on the respiratory and physical fitness status of acute post-COVID-19 patients. This randomized, single-blind, placebo-controlled study included 26 males (44 ± 17 years) and 24 females (38 ± 12 years), who performed a 6-min walking test (6 MWT) and pulmonary function test, specifically forced vital capacity (FVC) and expiratory volume in the first second (FEV1). Symptomatic participants were recruited between 21 and 33 days after a positive polymerase chain reaction test. The experiment consisted of H_2_/placebo inhalation, 2 × 60 min/day for 14 days. Results showed that H_2_ therapy, compared with placebo, significantly increased 6 MWT distance by 64 ± 39 m, FVC by 0.19 ± 0.24 L, and, in FEV1, by 0.11 ± 0.28 L (all *p* ≤ 0.025). In conclusion, H_2_ inhalation had beneficial health effects in terms of improved physical and respiratory function in acute post-COVID-19 patients. Therefore, H_2_ inhalation may represent a safe, effective approach for accelerating early function restoration in post-COVID-19 patients.

## 1. Introduction

Coronavirus disease 2019 (COVID-19) is a novel infectious disease caused by severe acute respiratory syndrome coronavirus 2 (SARS-CoV-2), which is responsible for the worldwide unpredictable pandemic situation. To date (9 November 2021), statistical data indicated ~250 million confirmed cases of COVID-19 and over 5 million deaths globally (https://ourworldindata.org, accessed on 9 November 2021). COVID-19 patients typically exhibit clinical symptoms such as a fever, headache, dry cough, shortness of breath, and severe fatigue [1,2]. Post-acute COVID-19 syndrome commonly manifests as a variety of persistent symptoms, such as severe fatigue, shortness of breath [3], headache, and attention disorder [4], that occur beyond 4 weeks from the onset of COVID-19 symptoms [5]. Recently, Mehta et al. [6] suggested that the residual abnormalities in health status after COVID-19 might, in part, be a consequence of the acute phase, pathological immune system response to ongoing infection known as the “cytokine storm”. In addition, it has recently been reported that viral infection induces an excessive proinflammatory response, including increased oxidative stress and apoptosis, which may be contributing factors to the etiology and pathogenesis of COVID-19 [7]. Similarly, Cumpstey et al. [8] described COVID-19 as a redox disease because an inflammation-driven “oxidative storm” alters the redox landscape, eliciting mitochondrial, metabolic, endothelial, and immune dysfunction. Importantly, Xu et al. [9] reported that augmented airway resistance, already associated with elevated proinflammatory interleukin-6 [10], may be considered a contributing factor that causes the increased mechanical work of breathing and leads to dyspnea and further COVID-19 progression.

From an impaired physical function standpoint, Paul et al. [11] found an interesting intersection of risk factors in patients with both COVID-19 and myalgic encephalomyelitis/chronic fatigue syndrome, particularly cell redox dysregulation, systemic inflammation, and an impaired ability to produce mitochondrial adenosine triphosphate (ATP) that all may be involved in post-acute COVID-19 syndrome, which is often accompanied by deteriorated physical exercise capacity [12]. Interestingly, Smith [13] formulated the “cytokine hypothesis of overtraining” more than 20 years ago, highlighting the negative role of elevated circulating proinflammatory cytokines (interleukin-1β, interleukin-6, tumor necrosis factor alpha) on whole body regulation, inducing “sickness” behavior and a decline in performance.

A change in physical function in post-COVID-19 patients has been assessed using the 6-min walking test (6 MWT) [14,15,16,17]. This test is a valid, reliable, and sensitive test for measuring changes in cardiorespiratory fitness in response to interventions [18] or post-COVID-19 rehabilitation [19], which is of great importance in the current post-pandemic era.

Molecular hydrogen (H_2_) has been shown to be a healthy, safe gas [20] with a strong and selective antioxidative capability for scavenging the harmful hydroxyl radical and peroxynitrite anion [20,21]. Numerous studies have indicated that H_2_ has anti-inflammatory [22], anti-apoptosis [23], antifatigue [24,25,26,27], and regulatory properties [28]. Based on the reported beneficial health effects across a variety of diagnoses [22,29], H_2_ administration has recently been proposed as a promising therapeutic gas for COVID-19 patients [7,30,31,32,33,34,35]. For instance, Guan et al. [36] showed clinically beneficial effects of a hydrogen/oxygen (H_2_–O_2_; 66–33%) mixed gas inhalation for the amelioration of most respiratory symptoms, such as dyspnea, chest distress, or cough, within days 2 and 3 of hospitalization for COVID-19 patients.

The aim of the study was to assess the effect of 14 days of H_2_ inhalation in patients with acute post-COVID-19 syndrome. Based on the aforementioned recent findings, we hypothesized that there would be a significant improvement in 6 MWT distance and respiratory function variables after 14 days of H_2_ inhalation.

## 2. Materials and Methods

### 2.1. Participants

**Figure 1:**
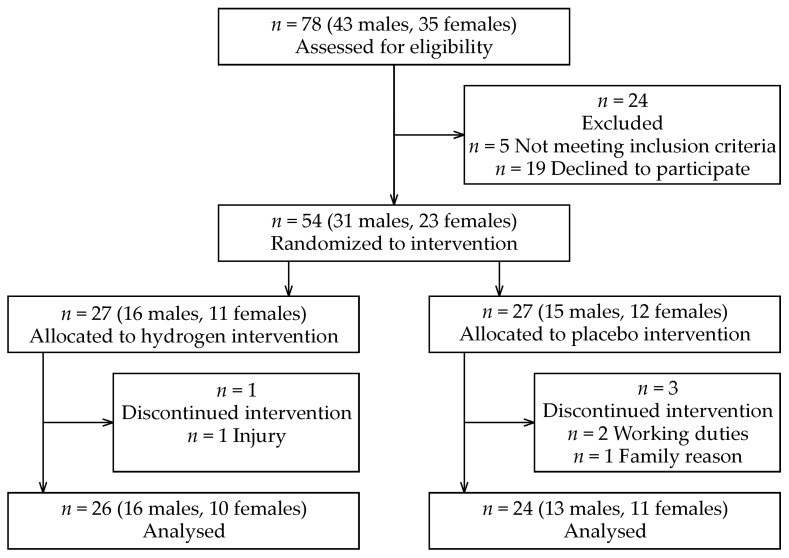
CONSORT flow diagram.

This parallel, single-blind, placebo-controlled study with block randomization included 26 males and 24 females (Figure 1), whose characteristics are presented in Table 1. Study participants were recruited using social networks and by collaborating medical professionals. Inclusion criteria were as follows: (1) age, 18–65 years; (2) with laboratory-confirmed SARS-CoV-2 infection using real-time reverse transcription polymerase chain reaction (RT-PCR) assay of nasal and pharyngeal swabs for COVID-19; (3) non-vaccinated and with manifestation of the self-reported clinical symptoms of COVID-19 (Table 2); (4) clinically stable to perform pre- and post-laboratory examinations; (5) without a resting oxygen saturation (SpO_2_) below 95%; and (6) having a positive RT-PCR test 21–35 days previously. Exclusion criteria were defined as: (1) hospitalization due to COVID-19; and (2) regular smoker. In addition, all participants only had COVID-19 and were free of other known (self-reported) cardiovascular, pulmonary, neurological, and metabolic diseases. The study was approved by the Ethics Committee of the Faculty of Physical Culture, Palacký University Olomouc, Olomouc, Czech Republic (protocol code 26/2021 and date of approval 28 February 2021). To the best of our knowledge, no side effects during or after the H_2_ application have been reported [29,37] or were reported in the present study.

### 2.2. Experimental Therapeutic Protocol

**Figure 2:**
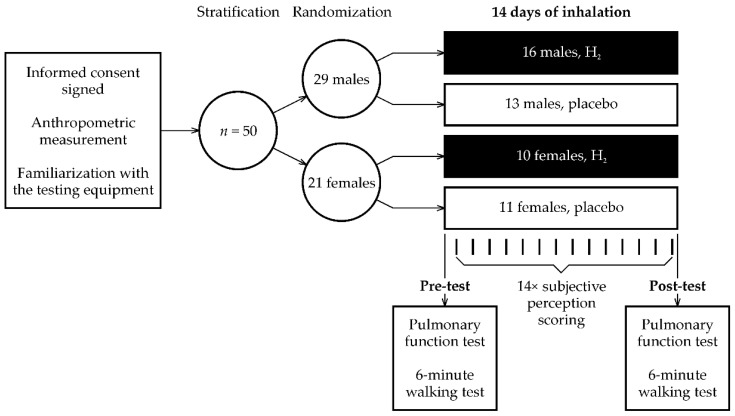
Overview of the study protocol.

The experimental therapeutic protocol (Figure 2) included pre- and post-therapeutic laboratory sessions interspersed by two weeks of home, self-administrated H_2_ inhalation. During the first session, participants were provided with the study information and familiarized with the testing laboratory equipment, and they also received instructions and training for safe operation of the H_2_ generator. They provided written informed consent in accordance with the Declaration of Helsinki. To assess the level of functional status impairment after COVID-19, participants were asked to complete the Post-COVID-19 Functional Status (PCFS) Scale [38]. Anthropometric measurements were then taken in the pre-examination only, whereas the pulmonary function and physical fitness tests were performed during the pre- and post-therapeutic sessions. The participants were advised to avoid drinking coffee, tea, and/or any other substance potentially affecting the selected physiological performance and perceptual responses to the function tests for at least two hours before both the pre- and post-therapeutic sessions. In addition, participants were also asked to avoid alcohol for 48 h before all pre- and post-laboratory testing. To avoid possible diurnal variations, all laboratory testing was scheduled between 8:30 and 11:00 AM in a faculty facility. Participants were randomly divided into H_2_ inhalation and placebo using a randomization table. The table was generated before the experiment using a random number generator (the randperm function available in MATLAB R2020a, MathWorks, Natick, MA, USA). Randomization used a block method to ensure a balance in sample size across subgroups and was stratified by sex.

### 2.3. Basis Anthropometric Measurement

Participant body height and body mass (to the nearest 0.1 kg) were measured using a digital weighing scale SOEHNLE 7307 (Leifheit, Nassau, Germany). Percent body fat was determined using bio-impedance analysis (Tanita MC-980MA, Tanita, Tokyo, Japan).

### 2.4. Pulmonary Function Testing

Each participant performed a standardized pulmonary function test on a spirometer (Ergostik, Geratherm Respiratory, Bad Kissingen, Germany) that was calibrated daily in accordance with the American Thoracic Society and European Respiratory Society technical statement [39]. The pulmonary function test was performed by the same technician. For each participant, the pre- and post-testing were performed at approximately the same time of day. The primary parameters assessed were as follows: forced vital capacity (FVC), forced expiratory volume in the first second (FEV1), and Tiffeneau index calculated as FEV1/FVC ratio. All variables were recorded during three test attempts, and the attempt with the highest FEV1 was used for the analysis. All values were expressed as a percentage of predicted normal values.

### 2.5. Physical Fitness, Perceived Exertion, and Dyspnea Assessment

In order to determine global physical functioning, a simple and self-paced 6 MWT was conducted [18]. Before the 6 MWT, each participant was instructed to walk as far as possible for 6 min, back and forth on a standardized 30-m track, marked by two cones, situated in an indoor gym facility. The achieved distance in 6 min was the primary outcome. To calculate the 6 MWT distance as a percentage of normative values, median values for the age range 18 to 80 years were taken from Dourado et al. [40]. For each participant, the appropriate median value was selected based on sex and age. The percentage was then calculated as 100% × 6 MWT distance/median value.

Arterial oxygen saturation (SpO_2_) was monitored by pulse oximetry (Onyx Vantage 9590, Nonin Medical, Plymouth, MN, USA) before and during the 6 MWT. The lowest achieved SpO_2_ value was recorded as the representative SpO_2_ response. Immediately after the 6 MWT, each participant provided a rating of perceived exertion (RPE) score on the 6–20-point Borg’s scale [41] and dyspnea level based on the modified Medical Research Council dyspnea scale (Grade 0, breathless only with strenuous exercise, to Grade 4, too breathless to leave the home) available in the Global Initiative for Chronic Obstructive Lung Disease report [42] on page 28.

### 2.6. Psychometric Variables Assessment

Participants were asked to score, on a 5-point scale, their morning perceptions of fatigue, muscle soreness, dyspnea, and insomnia (0—none to 4—severe). Scores were collected daily during the 14 days of the intervention. A 14-day average was calculated for subsequent statistical analysis.

### 2.7. Hydrogen/Placebo Inhalation Protocol

Participants inhaled, via a nasal cannula, either a 300 mL/min dose of H_2_ produced by the HB-H12 H_2_ generator (Guangzhou Hibon Eletronic Technology, Guangzhou, China) or placebo (ambient air) produced by a technically modified HB-H12 H_2_ generator (Leancat, Prague, Czech Republic). According to the operation manual, the H_2_ generator provides H_2_ at 99.99% purity, produced via purified water electrolysis using a membrane electrode assembly/proton exchange membrane. Inhalation of 100% H_2_ produced by a H_2_ generator through a nasal cannula, even at low flow rates (250 mL/min), was demonstrated to be an effective method of H_2_ administration [43]. Participants could not distinguish between the inhalation of H_2_ and placebo because H_2_ is colorless, odorless, and tasteless [29]. H_2_ or placebo were inhaled during two (morning and afternoon) 60-min home sessions under resting conditions. To our knowledge, there is a lack of studies from which the optimal duration of H_2_ inhalation for rehabilitation after COVID-19 can be derived. In general, rehabilitation after COVID-19 ranged from 5 days to 6 months [44]. In sports medicine, the duration of H_2_ administration prior to exercise ranged from 30 min to 4 weeks [45]. Therefore, we chose a 14-day H_2_/placebo intervention as a compromise to keep the duration long enough to reveal a detectable effect on physical and respiratory outcomes, yet acceptably short for study compliance.

### 2.8. Statistical Analysis

All data were recorded in Excel 365 (Microsoft, Redmond, WA, USA) tables for subsequent statistical processing. Data are presented as arithmetic mean and standard deviation or 95% confidence interval (CI). The normal distribution of variables was verified using the Kolmogorov–Smirnov test. An analysis of covariance (ANCOVA) with intervention factor (levels: H_2_ and placebo), sex factor (levels: male and female), and age as covariate was used to calculate the significances of the intervention and sex. An analysis of variance (ANOVA) with intervention and sex factors was used for the age variable. ANOVA and ANCOVA were used to evaluate baseline values obtained before interventions and to evaluate changes caused by interventions (change = post-intervention value minus pre-intervention baseline). In cases where sex factor was statistically insignificant, the male and female subgroups were merged. Differences between H_2_ inhalation and placebo were then evaluated using a two-sample *t*-test. The significance of the change value from zero was evaluated using a one-sample *t*-test. When the normal distribution of the variable was not met, nonparametric alternatives were used, namely: Kruskal–Wallis test, Mann–Whitney U test, and Wilcoxon test. The association between the 6 MWT change and the changes in respiratory variables (FVC, FEV1, and FEV1/FVC) was evaluated using the Pearson’s correlation coefficient. For all statistical tests, *p* < 0.05 was considered statistically significant. In addition to statistical significance, Cohen’s standardized difference was used. Statistical analyses were performed using MATLAB with Statistics Toolbox R2020a (MathWorks, Natick, MA, USA).

## 3. Results

Raw data are available in Table S1. Participant characteristics are shown in Table 1 and symptoms during COVID-19 infection are listed in Table 2. The types of medications received by the participants were as follows (frequency and relative frequency): NSAID-s: 10 (20%); antipyretics and analgesics: 6 (12%); supplements (vitamins and minerals): 5 (10%); antiallergics: 1 (2%); and anticoagulants: 2 (4%). The reported levels of functional status impairment according to the PCFS Scale were as follows (frequency and relative frequency): Grade 1–negligible functional limitations: 27 (54%); Grade 2–slight functional limitations: 20 (40%); Grade 3–moderate functional limitations: 3 (6%).

All variables displayed in Table 1 and Table 3, Table 4, Table 5 and Table 6 were evaluated for normal distribution using the Kolmogorov–Smirnov test. SpO_2_ at rest, SpO_2_ after 6 MWT, daily dyspnea, dyspnea after 6 MWT, and RPE were significantly (all *p* ≤ 0.015) different from the normal distribution and, therefore, these variables were analyzed using nonparametric tests. The remaining variables were not statistically significantly (all *p* ≥ 0.061) different from the normal distribution and were analyzed using ANOVA or ANCOVA.

Differences in age, body mass, body height, and days after PCR test between interventions (H_2_ versus placebo) were not significant (all *p* ≥ 0.056, Table 1). Although there were significant differences in BMI (*p* = 0.002) and body fat (*p* = 0.006), randomization can be considered successful because it is not possible to control all variables simultaneously. Significant (all *p* < 0.001) differences in body mass, body height, and body fat between the sexes are known anthropological differences between males and females.

A comparison of baseline values (before intervention) is shown in Table 3. No significant differences (all *p* ≥ 0.089) were found between the four subgroups using the Kruskal–Wallis test for SpO_2_ at rest, dyspnea after 6 MWT, SpO_2_ after 6 MWT, and RPE. It can, therefore, be concluded that there were no differences between the interventions (H_2_ versus placebo). ANCOVA did not reveal any significant (all *p* ≥ 0.42) intervention factor in the remaining variables studied. It can be concluded that there were no significant differences between the H_2_ subgroups and the placebo subgroups before the start of the interventions. The results did show that females had a significantly (*p* = 0.004) higher physical fitness expressed as 6 MWT (114.0 % on average) compared to males (106.9 %).

No significant (all *p* ≥ 0.49, Table 4) differences were found between the H_2_ subgroups and placebo subgroups for all self-reported perceptual variables averaged over 14 days of intervention.

An analysis of changes after 14 days of intervention is shown in Table 5. There were significant differences (all *p* ≤ 0.021) in FVC, FEV1, and 6 MWT between interventions. However, neither sex factor nor age factor were significant (all *p* ≥ 0.18) in any of the variables studied. This means that the responses to the interventions were not dependent on sex or age. Therefore, it was possible to merge both sexes into one group and remove the age factor. This new statistical analysis is provided in Table 6.

The most important finding in Table 6 is that 14 days of H_2_ inhalation provided an improvement of 64 m (95% CI: 48 to 80 m) in 6 MWT, which was significant from zero (*p* < 0.001). Placebo inhalation increased 6 MWT distance by 9 m (95% CI: −4 to 21 m), which was not significant (*p* = 0.15). The difference in improvement between H_2_ and placebo was significant (*p* < 0.001). RPE was significantly (*p* = 0.036) reduced by 0.9 points in the placebo group, but the decrease of 0.7 points was not significant (*p* = 0.11) in the H_2_ group. The difference between the interventions was not significant (*p* = 0.88). H_2_ inhalation also provided a 4.3% (95% CI: 2.0 to 6.6%) improvement in FVC, which was significant from zero (*p* = 0.001) and from placebo intervention (*p* = 0.005), which demonstrated no significant change (−0.2%, 95% CI: −2.4 to 2.2%, *p* = 0.85). The improvement in FEV1 after H_2_ inhalation was not significant (2.8%, 95% CI: −0.5 to 6.1%, *p* = 0.088) and the decrease after placebo inhalation was not significant (−2.2%, 95% CI: −5.3 to 1.0%, *p* = 0.17). However, the difference between interventions was significant (*p* = 0.028). No significant (*p* ≥ 0.42, Table 6) differences between interventions were found in the remaining studied variables.

**Figure 3:**
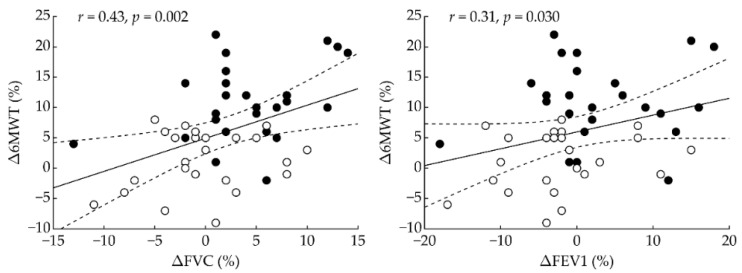
Correlation analysis between change in 6-min walking test and changes in respiratory variables. Δ—change between post-intervention and pre-intervention; 6 MWT—6-min walking test; FVC—forced vital capacity; FEV1—forced expiratory volume in the first second; *r* = Pearson’s correlation coefficient; *p* = statistical significance. Filled and open circles indicate participants who received H_2_ intervention and placebo, respectively. Dashed lines denote 95% confidence interval.

Correlation analysis (Figure 3) revealed significant correlations between FVC change and 6 MWT change (*r* = 0.43, *p* = 0.002) and between FEV1 change and 6 MWT change (*r* = 0.31, *p* = 0.030). The correlation between FEV1/FVC change and 6 MWT change (*r* = −0.02, *p* = 0.91) was not significant.

## 4. Discussion

To the best of our knowledge, this is the first randomized, placebo-controlled study to examine whether home-based H_2_ inhalation therapy (2 × 60 min/day, for 14 days) could improve respiratory and physical function during early recovery in acute post-COVID-19 patients. The main findings of this novel study are as follows: H_2_ inhalation compared to placebo induced an (1) increase in 6 MWT distance (H_2_: 64 ± 39 m, placebo: 9 ± 29 m, *p* < 0.001); (2) increase in FVC (H_2_: 0.19 ± 0.24 L, placebo: −0.01 ± 0.22 L, *p* = 0.004); (3) increase in FEV1 (H_2_: 0.11 ± 0.28 L, placebo: −0.08 ± 0.27 L, *p* = 0.025); and (4) improvements in FVC (*r* = 0.43, *p* = 0.002) and FEV1 (*r* = 0.31, *p* = 0.030) that correlated significantly with improvement in 6 MWT.

There is a growing body of evidence that physical function is impaired following both COVID-19 [12,16] and severe acute respiratory syndrome (SARS) [46] that persists for several weeks or months post-infection. It has been well documented that a sedentary lifestyle is generally associated with lower physical fitness [47]. In this regard, a considerable reduction in the amount of physical activity due to quarantine and social contact restrictions, due to the COVID-19 pandemic [48], may have a negative deconditioning effect on physical functioning that is similar to the effects of a sedentary lifestyle in COVID-19 patients. The 6 MWT is widely accepted as “a gold standard” for cardiorespiratory capacity, primarily in patients with chronic respiratory disease [18], and has been considered as an appropriate test to triage COVID-19 patients [14]. Our results showed that pre-intervention distance covered during the 6 MWT was 679 m (107%) for males and 666 m (114%) for females according to reference values adjusted for age and sex [40]. Our cohort of acute post-COVID-19 participants exhibited generally good physical function, despite still experiencing persisting symptoms, such as fatigue, dyspnea, or muscle soreness (Table 4), up to 26 days, on average, after a positive PCR test. Townsend et al. [49], who assessed patients aged ~50 years and with greater COVID-19 severity, reported a 6 MWT distance of ~460 m, which was below the healthy population performance level [50]. Surprisingly, the 6 MWT result was not associated with either initial disease severity or respiratory complications after 75 days of diagnosis [49]. On the other hand, Blanco et al. [51] reported a significantly better result for the 6 MWT (~577 m) in older patients (~55 years old) with less severe COVID-19 up to 104 days after the onset of symptoms. In another study, Baranauskas et al. [52] found no significant differences in physical function between post-COVID-19 patients and the control group; however, the post-COVID-19 patients had impaired postexercise autonomic cardiac regulation up to 3 months after diagnosis. Based on our results and the above evidence from the literature, deteriorated post-COVID-19 physical function tends to improve a few weeks or months after the onset of symptoms, but residual health abnormalities associated with infection may still persist.

Impaired physical fitness, as well as long-lasting fatigue, during post-acute COVID-19 phase may have a common denominator—oxidative stress. Coronavirus induced oxidative stress and its related negative consequences on cellular homeostasis, including a redox dysbalance, and deteriorated mitochondrial functions and ATP productions [8,11,53], which have long been associated with both fatigue [54] and with decline in physical fitness [55]. In this context, H_2_ has repeatedly been considered a strong selective antioxidant [20,21] with the ability to protect mitochondrial respiratory function and ATP production [20,56,57], as well as being a suitable agent for the treatment of temporary and chronic forms of oxidative-stress-associated fatigue [58]. The most important finding of the present study is that 14 days of H_2_ inhalation, performed at home, resulted in an improvement in physical function compared to the placebo group, irrespective of sex and age. Specifically, the distance covered during the 6 MWT was extended by 64 m after H_2_ therapy, whereas there was only a 9 m increase in the placebo group. An increased distance of 30 m for the 6 MWT has previously been established as the minimal clinically important improvement in adults with chronic respiratory diseases [18]. Hence, we suggest that 2 weeks of daily H_2_ inhalation resulted in a clinically relevant improvement in physical function in our cohort of acute post-COVID-19 patients. From an improved physical fitness standpoint, the antifatigue effect of H_2_ demonstrated in the present study has already been documented in other studies examining different modes of exercise in a healthy population [26,27], well-trained athletes [25,45,59], and animal models [24]. The antifatigue effect of H_2_ supplementation was explained by its ability to stimulate oxidative metabolism, reduce oxidative stress, adjust the cellular redox environment and improve immune function. Interestingly, the improvements in 6 MWT distance and in the respiratory variables were independent of sex and age. It appears that the law of initial values did not play a role here. If the law of initial values were valid, then the improvement should depend on the pretest value and, therefore, on age, because the 6 MWT distance, FVC, and FEV1 were age-dependent (Table 3). However, this result should be interpreted with caution as it may be due to insufficient sample size. In addition, the changes after 14 days of H_2_ inhalation may be dependent on the severity of COVID-19. Therefore, further studies with a larger sample size stratified by COVID-19 severity are needed to verify this result.

A second important finding in the present study was the similar RPE level in both groups in response to the post-intervention 6 MWT. However, only the H_2_ group demonstrated a clinically relevant improvement in distance walked. In this situation, one would expect that a faster walking pace would be associated with a higher RPE. Borg’s RPE has traditionally been interpreted as reflecting a complex feedback mechanism that is modulated by a variety of physiological functions, including HR rhythm, minute ventilation and breathing frequency, muscle and joint stiffness, and central fatigue [60]. Therefore, we suggest that daily H_2_ inhalation could induce a higher perceived tolerability (resistance) to increased walking pace in our acute post-COVID-19 patients. In addition, our results show that H_2_ gas inhalation had a beneficial effect on respiratory function, and the H_2_-induced improvement in FVC was associated with gain in cardiorespiratory capacity. We propose that the positive functional changes induced by H_2_ inhalation may be attributed to the higher perceived tolerability to the cardiorespiratory test in our participants. An increased tolerability to high exercise intensity was previously reported by Botek et al. [61], who found a lower lactate response and improved ventilatory efficiency after pre-exercise H_2_ application.

Health benefits associated with H_2_ inhalation in hospitalized patients have recently been published by Guan et al. [36], who applied 6 L/min of H_2_–O_2_ (66%–33%) in an experimental group of COVID-19 patients and a similar dose of O_2_ in a control group. H_2_–O_2_ inhalation resulted in a significantly reduced disease severity, including reduced dyspnea, coughing, chest distress, and pain. Improvements were rapid and were demonstrated after the second and third days, as well as at the end of the treatment, compared to the control group. The clinical benefits of H_2_–O_2_ administration have been attributed to the ability to reduce inspiratory efforts due to a considerably lower resistance to air when passing through the respiratory tract [62]. Lau et al. [63] showed that 6 weeks of a well-supervised exercise training program in ~40-year-old patients recovering from SARS induced a significant improvement in the 6 MWT distance of 77 m (baseline distance 590 m). This improvement in walking distance is almost the same as our result; however, H_2_ therapy is potentially a threefold more time-efficient rehabilitation approach than exercise training when it comes to improving 6 MWT performance for acute post-COVID-19 patients.

We feel that a combination of H_2_ administration with well-established post-COVID-19 rehabilitation programs [12,64] may have a synergistic rehabilitation effect, resulting in an enhanced restoration of physical and respiratory functions, and, subsequently, provide a faster return to normal life. Therefore, studies investigating the combination of H_2_ administration with other rehabilitation programs would be important future work. H_2_ administration seems to be a healthy, safe [20,29,65], well-tolerated therapeutic approach with no clinically significant health issues reported in animal model [37,43]. Therefore, we assume that H_2_ could be potentially applied at health rehabilitation facilities (spa), post-COVID-19 care units, or during telerehabilitation in post-COVID-19 patients.

This study has the following limitations: (1) for logistical reasons, there was only single blinding and, therefore, detection bias cannot be ruled out. (2) Morning perceptual measures were obtained from the participants, which could have resulted in self-reporting bias.

## 5. Conclusions

Our results suggest that 14 days of regular H_2_ inhalation may be considered as an efficient rehabilitation approach for improving both physical and respiratory function in acute post-COVID-19 patients.

## Figures and Tables

**Table 1:** Characteristics of participants.

	Male	Male	Female	Female	ANOVA/ANCOVA		
	H_2_	Placebo	H_2_	Placebo	Int.	Sex	Age
	Mean ± SD	Mean ± SD	Mean ± SD	Mean ± SD	*p*	*p*	*p*
*n* = 50	16	13	10	11			
Age (years)	45 ± 19	39 ± 11	41 ± 13	37 ± 12	0.22	0.48	-
Body mass (kg)	82.7 ± 9.2	76.7 ± 9.3	69.1 ± 12.1	62.5 ± 7.1	0.056	<0.001	0.007
Body height (cm)	179.3 ± 6.6	181.3 ± 8.1	167.6 ± 7.2	169.1 ± 7.2	0.44	<0.001	0.63
BMI (kg/m^2^)	25.7 ± 2.4	23.4 ± 2.5	24.5 ± 3.0	21.8 ± 2.0	0.002	0.078	<0.001
Body fat (%)	18.2 ± 6.7	14.3 ± 4.8	30.5 ± 7.3	22.5 ± 6.4	0.006	<0.001	<0.001
Days after PCR	26.6 ± 4.1	24.7 ± 4.1	26.4 ± 3.7	26.1 ± 4.3	0.28	0.65	0.82

ANOVA—analysis of variance with factors intervention and sex; ANCOVA—analysis of covariance with factors intervention, sex, and age; H_2_—molecular hydrogen; Int.—intervention; SD—standard deviation; *p*—statistical significance; BMI—body mass index; PCR—polymerase chain reaction test.

**Table 2:** List of symptoms of coronavirus disease 2019 (COVID-19) in study group of 50 participants.

Symptom	Frequency	Relative Frequency
Anxiety	1	2%
Cognitive impairment	2	4%
Cough	8	16%
Diarrhea	1	2%
Dyspnea	38	76%
Fatigue	40	80%
Fever	28	56%
Headache	19	38%
Insomnia	15	30%
Joint/muscle aches	20	40%
Loss of taste/smell	17	34%
Shiver	1	2%
Sore throat	3	6%

**Table 3:** Baseline values of spirometry and 6-min walking test.

	Male	Male	Female	Female	ANCOVA			K-W
	H_2_	Placebo	H_2_	Placebo	Int.	Sex	Age	
	Mean ± SD	Mean ± SD	Mean ± SD	Mean ± SD	*p*	*p*	*p*	*p*
FVC (L)	4.92 ± 1.01	5.22 ± 0.68	3.61 ± 0.72	3.85 ± 0.61	0.51	<0.001	<0.001	
FVC (%)	96.7 ± 14.6	99.8 ± 12.0	106.5 ± 11.3	108.5 ± 11.3	0.55	0.017	0.55	
FEV1 (L)	4.11 ± 1.01	4.45 ± 0.54	2.94 ± 0.70	3.18 ± 0.46	0.42	<0.001	<0.001	
FEV1 (%)	103.9 ± 17.9	107.5 ± 14.1	100.9 ± 18.5	104.1 ± 10.5	0.55	0.43	0.38	
FEV1/VC	0.831 ± 0.075	0.856 ± 0.064	0.813 ± 0.094	0.830 ± 0.063	0.54	0.19	0.017	
SpO_2_rest (%)	97.5 ± 0.8	98.0 ± 0.7	98.3 ± 0.7	98.0 ± 1.0				0.089
Dyspnea (points)	1.3 ± 0.6	1.3 ± 0.6	1.2 ± 0.6	1.5 ± 0.5				0.61
6 MWT (m)	671 ± 80	689 ± 27	654 ± 62	676 ± 36	0.60	0.095	<0.001	
6 MWT (%)	106.6 ± 8.9	107.2 ± 5.1	113.3 ± 10.1	114.7 ± 8.3	0.69	0.004	0.89	
SpO_2_walk (%)	94.1 ± 2.3	94.6 ± 3.0	94.6 ± 2.6	94.7 ± 4.1				0.71
RPE (points)	12.2 ± 1.8	11.7 ± 1.8	11.4 ± 1.4	12.2 ± 1.8				0.65

ANCOVA—analysis of covariance with factors intervention, sex, and age; K-W—Kruskal–Wallis test; H_2_—molecular hydrogen; Int.—intervention; SD—standard deviation; *p*—statistical significance; FVC—forced vital capacity; FEV1—forced expiratory volume in the first second; SpO_2_rest—oxygen saturation in resting condition; 6 MWT—6-min walking test; SpO_2_walk—oxygen saturation during 6-min walking test; RPE—rate of perceived exertion.

**Table 4:** Average subjective perceptions of fatigue, sleep quality, muscle soreness, and dyspnea during 14 days of intervention.

	Male	Male	Female	Female	ANCOVA			K-W
	H_2_	Placebo	H_2_	Placebo	Int.	Sex	Age	
	Mean ± SD	Mean ± SD	Mean ± SD	Mean ± SD	*p*	*p*	*p*	*p*
Fatigue	1.9 ± 0.6	1.9 ± 0.6	2.1 ± 0.7	2.1 ± 0.5	0.81	0.20	0.18	
Sleep quality	1.6 ± 0.9	1.6 ± 0.8	1.6 ± 0.6	1.6 ± 0.9	0.49	0.63	0.002	
Muscle soreness	1.5 ± 0.5	1.5 ± 0.5	1.4 ± 0.5	1.5 ± 0.4	0.80	0.99	0.80	
Dyspnea	0.6 ± 0.5	0.5 ± 0.5	0.5 ± 0.5	0.6 ± 0.6				0.85

ANCOVA—analysis of covariance with factors intervention, sex, and age; K-W—Kruskal–Wallis test; H_2_—molecular hydrogen; Int.—intervention; SD—standard deviation; *p*—statistical significance. Values of subjective perceptions were recorded each day during 14 days of intervention and were averaged separately for each participant.

**Table 5:** Changes after 14 days of intervention in spirometry and 6-min walking test.

	Male	Male	Female	Female	ANCOVA			K-W
	H_2_	Placebo	H_2_	Placebo	Int.	Sex	Age	
	Mean ± SD	Mean ± SD	Mean ± SD	Mean ± SD	*p*	*p*	*p*	*p*
FVC (L)	0.19 ± 0.29	0.00 ± 0.22	0.19 ± 0.15	−0.02 ± 0.23	0.003	0.78	0.18	
FVC (%)	3.6 ± 6.4	−0.1 ± 4.4	5.4 ± 4.2	−0.4 ± 6.3	0.003	0.73	0.19	
FEV1 (L)	0.08 ± 0.33	−0.09 ± 0.27	0.15 ± 0.19	−0.05 ± 0.28	0.021	0.54	0.58	
FEV1 (%)	1.5 ± 8.9	−2.5 ± 6.7	5.0 ± 6.7	−1.8 ± 8.6	0.020	0.41	0.43	
FEV1/VC	−0.015 ± 0.048	−0.017 ± 0.036	−0.002 ± 0.041	−0.011 ± 0.038	0.71	0.40	0.74	
SpO_2_rest (%)	0.3 ± 0.8	0.2 ± 0.4	0.1 ± 0.6	0.2 ± 1.0				0.70
Dyspnea (points)	−0.9 ± 0.8	−0.8 ± 0.4	−0.7 ± 0.9	−0.6 ± 0.7				0.64
6 MWT (m)	65 ± 44	20 ± 28	62 ± 33	−5 ± 26	<0.001	0.18	0.86	
6 MWT (%)	10.5 ± 7.2	3.2 ± 4.4	10.6 ± 5.4	−0.9 ± 4.7	<0.001	0.27	0.53	
SpO_2_walk (%)	1.4 ± 2.2	0.8 ± 2.6	1.8 ± 3.5	1.6 ± 2.8				0.75
RPE (points)	−0.8 ± 3.1	−0.8 ± 2.0	−0.4 ± 1.6	−1.0 ± 2.1				0.89

ANCOVA—analysis of covariance with factors intervention, sex, and age; K-W—Kruskal–Wallis test; H_2_—molecular hydrogen; Int.—intervention; SD—standard deviation; *p*—statistical significance; FVC—forced vital capacity; FEV1—forced expiratory volume in the first second; SpO_2_rest—oxygen saturation in resting condition; 6 MWT—6-min walking test; SpO_2_walk—oxygen saturation during 6-min walking test; RPE—rate of perceived exertion. Change was expressed as post-intervention value minus pre-intervention baseline.

**Table 6:** Changes after 14 days of intervention in spirometry and 6-min walking test, with merged subgroups of males and females.

	H_2_		Placebo					
	Mean ± SD	95% CI	Mean ± SD	95% CI	*d*	*p*	*p*1	*p*2
FVC (L)	0.19 ± 0.24	0.09 to 0.29	−0.01 ± 0.22	−0.10 to 0.08	0.85	0.004	0.001	0.83
FVC (%)	4.3 ± 5.7	2.0 to 6.6	−0.2 ± 5.2	−2.4 to 2.0	0.83	0.005	0.001	0.85
FEV1 (L)	0.11 ± 0.28	−0.01 to 0.22	−0.08 ± 0.27	−0.19 to 0.04	0.66	0.025	0.070	0.18
FEV1 (%)	2.8 ± 8.2	−0.5 to 6.1	−2.2 ± 7.5	−5.3 to 1.0	0.64	0.028	0.088	0.17
FEV1/VC	−0.010 ± 0.045	−0.028 to 0.008	−0.015 ± 0.036	−0.030 to 0.001	0.11	0.70	0.26	0.060
SpO_2_rest (%) *	0.2 ± 0.7	−0.1 to 0.5	0.2 ± 0.7	−0.1 to 0.5	−0.02	0.63	0.27	0.25
Dyspnea (points) *	−0.8 ± 0.8	−1.2 to −0.5	−0.8 ± 0.5	−1.0 to −0.5	−0.08	0.83	0.001	<0.001
6 MWT (m)	64 ± 39	48 to 80	9 ± 29	−4 to 21	1.58	<0.001	<0.001	0.15
6 MWT (%)	10.5 ± 6.4	7.9 to 13.1	1.3 ± 4.9	−0.8 to 3.4	1.61	<0.001	<0.001	0.21
SpO_2_walk (%) *	1.5 ± 2.7	0.5 to 2.6	1.2 ± 2.7	0.1 to 2.3	0.12	0.42	0.003	0.047
RPE (points) *	−0.7 ± 2.6	−1.7 to 0.4	−0.9 ± 2.0	−1.8 to −0.1	0.11	0.88	0.11	0.036

H_2_—molecular hydrogen; SD—standard deviation; CI—confidence interval; *d*—Cohen’s d; *p*—statistical significance between H_2_ and placebo (two-sample *t*-test or Mann–Whitney U test); *p*1—statistical significance of H_2_ to zero (one-sample *t*-test or Wilcoxon test); *p*2—statistical significance of placebo to zero (one-sample *t*-test or Wilcoxon test); FVC—forced vital capacity; FEV1—forced expiratory volume in the first second; SpO_2_rest—oxygen saturation in resting condition; 6 MWT—6-min walking test; SpO_2_walk—oxygen saturation during 6-min walking test; RPE—rate of perceived exertion; *—variables with a distribution statistically different from the normal distribution for which nonparametric tests were used.

## Data Availability

The data presented in this study are available in the Supplementary Materials.

## Supplementary Materials

The following supporting information can be downloaded at: https://www.mdpi.com/article/10.3390/ijerph19041992/s1, Table S1: Raw data.
